# Role of the interface between distributed fibre optic strain sensor and soil in ground deformation measurement

**DOI:** 10.1038/srep36469

**Published:** 2016-11-09

**Authors:** Cheng-Cheng Zhang, Hong-Hu Zhu, Bin Shi

**Affiliations:** 1 School of Earth Sciences and Engineering, Nanjing University, Nanjing 210023, China; 2 Department of Engineering, University of Cambridge, Cambridge CB2 1PZ, United Kingdom

## Abstract

Recently the distributed fibre optic strain sensing (DFOSS) technique has been applied to monitor deformations of various earth structures. However, the reliability of soil deformation measurements remains unclear. Here we present an integrated DFOSS- and photogrammetry-based test study on the deformation behaviour of a soil foundation model to highlight the role of strain sensing fibre–soil interface in DFOSS-based geotechnical monitoring. Then we investigate how the fibre–soil interfacial behaviour is influenced by environmental changes, and how the strain distribution along the fibre evolves during progressive interface failure. We observe that the fibre–soil interfacial bond is tightened and the measurement range of the fibre is extended under high densities or low water contents of soil. The plastic zone gradually occupies the whole fibre length when the soil deformation accumulates. Consequently, we derive a theoretical model to simulate the fibre–soil interfacial behaviour throughout the progressive failure process, which accords well with the experimental results. On this basis, we further propose that the reliability of measured strain can be determined by estimating the stress state of the fibre–soil interface. These findings may have important implications for interpreting and evaluating fibre optic strain measurements, and implementing reliable DFOSS-based geotechnical instrumentation.

Soils cover most of the land surface of Earth. A scientific measurement and characterization of the spatio-temporal deformations of geo-materials is critical to the understanding of earth surface processes and the safety of civil infrastructures during and after construction. However, conventional methods for measuring soil deformation, such as inclinometers, Global Positioning System (GPS) and surveying techniques, have remained quite limited in terms of either measurement range or accuracy^1^. The distributed fibre optic strain sensing (DFOSS) technique provides an elegant solution to this problem by enabling distributed strain measurement along an optical fibre over dozens of kilometres while maintaining a relatively high spatial resolution^2^,^3^,^4^,^5^,^6^,^7^,^8^. Recently this technique has been applied to measure deformations of a variety of earth structures such as slopes^1^,^9^,^10^,^11^,^12^,^13^,^14^, foundations^15^,^16^ and cavities and sinkholes^17^,^18^,^19^.

A reliable and effective DFOSS-based geotechnical monitoring scheme is conditioned by many factors. Although standard optical fibres as sensing elements are inexpensive, the cost of demodulators is quite substantial, which greatly hinders the widespread application of this technology. In addition, it is required that the strain sensing fibres can not only survive in harsh environments but also ensure the strain transfer from the surrounding geo-materials to the fibre core. Demodulators are also required to survive in highly aggressive environments because the quality of measurements may be degraded due to sunlight exposure, storm or severe cold. Moreover, special attention should be paid to the sensor installation process since poor-quality sensor installation and temperature compensation may bring uncertainties to the fibre optic measurements. Furthermore, setting of proper instrument parameters such as spatial resolution and sampling interval is key to improve the quality of data interpretation. Among all these factors, the effective attachment of optical fibres to soil masses has been considered to be one of the major barriers to the successful application of DFOSS technique to soil deformation measurement^20^. Unlike the monitoring of steel, concrete or composite structures^21^,^22^,^23^,^24^, for earth structures, optical fibres cannot be adhered directly to soils; hence, an intimate contact between a bare optical fibre and the surrounding soil mass can hardly be ensured. Further, soils are loose porous media and are particularly susceptible to the surrounding environment. Environmental changes, such as seasonal fluctuation of groundwater, drought, and rainfall infiltration, bring more uncertainties to the fibre–soil interfacial behaviour, thereby also affecting the validity of measured data.

Over the past decade, many researchers have attempted to detect the movements of earth structures by directly embedding optical fibres into soil masses^13^,^14^,^25^,^26^. The interaction between optical fibre and soil has therefore attracted considerable interest^11^,^19^,^27^,^28^,^29^,^30^. Recently an integrated DFOSS- and photogrammetry-based performance evaluation of a small-scale model of sand foundation under surcharge loads has brought our attention to this issue (Supplementary Fig. S1, and Fig. 1). Under plane strain conditions, the strains of soil along m–m’ were obtained by using soil-embedded optical fibres as well as photogrammetry and particle image velocimetry (PIV) techniques (Supplementary Fig. S1). Interestingly, while the strain distribution pattern of soil mass obtained by the two methods was similar, the strain magnitudes measured by the DFOSS system were much smaller, and the difference became more pronounced under higher surcharge load, which induced larger horizontal displacements of soil at the installation locations of the optical fibres (Fig. 1). Although there are many factors that can explain this discrepancy, the deformation compatibility between optical fibres and soil has been considered to be a dominant factor^14^,^25^,^26^. In this aspect, a pioneer study reported by Iten *et al*.^11^ suggested that pull-out tests can be an effective method to understand the interaction between optical fibre and soil. Subsequent research performed by our group has shown that the overburden pressure (OP) can effectively tighten the fibre–soil interfacial bond, thereby extending the measurement range of the optical fibre^27^,^28^,^29^. However, this phenomenon was discovered in pull-out tests on small soil samples and the strain distribution along the optical fibre was not obtained during the failure process of fibre–soil interface. In addition, the physical and mechanical properties of natural soils (e.g. water content, density and strength) can easily be influenced by complicated geological and environmental conditions, which may result in undesirable deterioration of the fibre–soil interface. However, up to now, no detailed investigation has been presented to elucidate the influence of these factors on the fibre–soil interfacial behaviour.

Here we examine the influence of environmental changes on the fibre–soil interfacial bond and the measurement range of optical fibre. We also examine the evolution of the distribution of strain along the optical fibre during the interfacial failure process. In addition, we present a theoretical model that can accurately describe and predict the fibre–soil interfacial behaviour throughout the whole deformation process of soil. On this basis, we further propose that the reliability of measured fibre optic strain data can be determined by estimating the stress state of the fibre–soil interface.

## Results

### Fibre–soil interfacial characteristics are susceptible to environmental changes

We previously showed that OP is a critical factor that affects the fibre–soil interfacial properties^27^,^28^,^29^. Here we focused on the influence of density and water content of soil. Typical pull-out force–displacement curves under various dry densities and water contents of soil are shown in Supplementary Fig. S2. Peak and residual interfacial shear strengths (ISSs) increased with dry density of soil (Fig. 2a, and Supplementary Fig. S3a), indicating that under high soil densities, the fibre–soil interfacial bond is enhanced significantly. Consistent with this, increases in dry densities of soil were accompanied by the increase of effective and residual displacements (Fig. 2b, and Supplementary Fig. S3b). This suggests that the measurement range of optical fibre is extended under high densities of soil. Conversely, increasing water contents of soil caused reductions of peak and residual ISSs, and effective and residual displacements (Fig. 2f,g, and Supplementary Fig. S3c,d). Notably, this trend was not affected by the optimum water content of soil. This observation indicates that water content of soil plays an utterly negative role in the fibre–soil interfacial bond.

The ratio of residual to peak ISS has been considered to be an important indicator in soil deformation monitoring as this parameter helps us identify whether the optical fibre is in a valid working state from a field monitoring standpoint^28^,^29^. We previously demonstrated that the ratio of residual to peak ISS was not correlated with OP while the interfacial bond was highly sensitive to the OP^29^. Not unexpectedly, the ratio of residual to peak ISS appeared to be dry density- and water content-independent as well (Fig. 2c,h). However, this observation requires further dedicated studies.

The ISS–OP relationships under different dry densities and water contents of soil were well fitted by linear lines (Supplementary Fig. S4). This indicates that the fibre–soil interface obeys the well-known Mohr–Coulomb failure criterion. The fitted lines were used to obtain the cohesions and friction angles of the fibre–soil interface (Supplementary Table S1). Consistent with ISSs, the cohesions and friction angles increased with dry density of soil, while the increase in water contents of soil caused reductions of these two parameters. Collectively, our results indicate that the fibre–soil interfacial characteristics are susceptible to the overburden pressure, density and water content of soil.

### Measuring strain distribution evolution during progressive fibre–soil interface failure

Next we sought to investigate the evolution of strain distribution along the optical fibre during the interfacial failure process. A 1200-mm long and 2-mm diameter optical fibre was tested in a sand-filled tank (Supplementary Fig. S5), where the fibre optic strain data were obtained using a Brillouin optical time-domain analysis (BOTDA) demodulator with a spatial resolution of 50 mm and a sampling interval of 10 mm, allowing for the acquisition of 100 data points over a 1-m distance. Detailed experiment procedure is described in the “Materials and Methods” section. The experimental results are shown in Fig. 3. Applied pull-out forces measured by a force gauge (*P*_FG_) agreed well with those calculated using the fibre optic strain values at the fibre head (*P*_FOS_) (Fig. 3b), indicating the experimental setup was reliable and the measured fibre optic strain data were accurate. The relationship between pull-out force and pull-out displacement displays some important features (Fig. 3a). Up to peak, the pull-out force increased with an increasing displacement and the curve was highly non-linear. Differently, the pull-out force tended to be stable after peak in spite of the continuous increase of the displacement.

Axial strains of the fibre were obtained under 12 displacement steps (Fig. 3c). Remarkably, the strains emerged at the loading point and then propagated towards the far end of the fibre with the increase of pull-out displacement. The strains were fully mobilised at a displacement of 5.36 mm, corresponding to a pull-out force of 10.8 N. Prior to that, the mobilised length was correlated with the pull-out force and their relationship curve was fitted using a linear line (Fig. 3d). Additionally, two distinctive zones were identified within the mobilised length. The strain distribution in the first zone can be approximated by a straight line, whereas that in the remaining portion can be fitted by a non-linear curve. The straight portion increased with the applied displacement and eventually distributed along the whole fibre length. Importantly, because differentiation of axial strain with respect to position may yield ISS, our results show that the plastic or the failure zone, characterized by a constant value of ISS, propagated towards the fibre toe with the increase of pull-out displacement, and finally occupied the whole fibre length. Combined with the relationship between pull-out force and pull-out displacement, these observations indicate that during the progressive failure process the fibre–soil interfacial behaviour can be described by using an ideal elasto–plastic model.

### Interpretation of the experimental results using a simplified fibre–soil interaction model

Here we proposed a simplified model to describe and interpret the fibre–soil interfacial behaviour during the progressive failure process (Fig. 4). We assumed an elasto–plastic shear stress–strain constitutive relations for the fibre–soil interface:


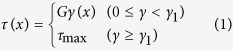


where *τ* and *γ* are the interfacial shear stress and strain, respectively; *γ*_1_ is the interfacial shear strain corresponding to the peak ISS, *τ*_max_; and *G* is the shear stiffness of the fibre–soil interface. The curve has an elastic branch up to the peak ISS (denoting a perfectly coupled interface), followed by a horizontal branch indicating the fibre–soil interface is totally decoupled (Fig. 4a).

With the increase of the pull-out force applied on the fibre head, the ISS and the axial strain increases gradually. At first no interface debonding occurs (Fig. 4b, left panel). Once the ISS at the fibre head reaches *τ*_max_, interface debonding initiates and propagates from the fibre head to the toe. At this stage, both elastic and plastic stress states of the interface exist along the optical fibre (Fig. 4b, middle panel). When the ISS peaks at the fibre toe, the interface is entirely debonded (Fig. 4b, right panel). We therefore divided the whole pull-out process into three phases (Fig. 4c), i.e. the pure elastic phase (Phase I), the elasto–plastic phase (Phase II) and the pure plastic phase (Phase III). We derived the relation between pull-out force *P* and pull-out displacement *u*_0_ for each pull-out phase as


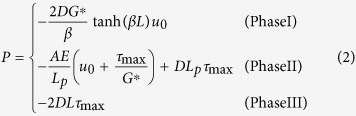


where *D*, *L*, *A* and *E* are the diameter, length, cross-sectional area and Young’s modulus of the optical fibre, respectively; G^*^ is a shear coefficient of the fibre–soil interface defined as G^*^=2*G*/*h*; *h* is the thickness of the shearing band along the fibre; *β* is a coefficient defined by 
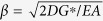
; and *L*_*p*_ is the length of the plastic zone. In addition, we also obtained the distributions of axial strain, interfacial shear stress, and displacement along the optical fibre for each of the three pull-out phases. Detailed formulations are provided in the Supplementary Information.

The model was employed to simulate the experimental results using the following parameters: *D* = 2 mm, *L* = 1.2 m, *E* = 0.34 GPa, G^*^ = 20 MPa/m, and *τ*_max_ = 1.53 kPa. The former two parameters (*D* and *L*) were the actual dimensions of the fibre. The Young’s modulus *E* of the fibre was obtained using a series of standard uniaxial tensile tests. The latter two parameters (G^*^ and *τ*_max_) were obtained by fitting the proposed model to the experimental data using a least squares method. The predicted results obtained by simulation agreed reasonably well with the experimental data (Fig. 3a,c, red dashed lines). Notably, the axial strain, with respect to distance, is a hyperbolic function and a linear function in the elastic zone and the plastic zone, respectively (see equations (S6) and (S10) in Supplementary Information), which is consistent with our previous description of the two distinctive zones of the strain distribution. Thus, our model can rationally describe the progressive failure behaviour of the fibre–soil interface, and accurately predict the evolution of the strain distribution.

On this basis, we further proposed that three working states of a strain sensing fibre can be identified during the course of fibre–soil interface failure, namely the effective state, the partially effective state and the invalid state (Fig. 4b). Under the effective working state, the fibre–soil interface is at the elastic stress state and the strain measurements are considered as reliable. Conversely, under the invalid working state, the interface is at the plastic stress state and the measured data should be regarded as invalid. For a specific segment of an optical fibre, the reliability of strain measurements can be determined according to the interface working state of that segment, which can be estimated using a simple approach in engineering practice^27^,^30^. In this approach, the ISS can be expressed as


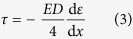


where d*ε*/d*x* is the gradient of strain along the fibre segment. If the calculated ISS *τ* is smaller than the peak ISS *τ*_max_, the measured strain data can be regarded as valid.

## Discussion

This paper highlights the role of distributed fibre optic strain sensor–soil interface in measuring soil deformation. We elucidated the influence of environmental changes on the fibre–soil interfacial behaviour, examined the evolution of strain distribution along the optical fibre during progressive interface failure, and derived a theoretical model to simulate the experimental data. These results led to a simple approach to evaluate the reliability of measured fibre optic strain data. To our knowledge, this is the first comprehensive characterization of the fibre–soil interface for application of DFOSS technique to soil deformation measurement.

Previous studies have reported overburden pressure as an important role in the fibre–soil interfacial behaviour^27^,^28^,^29^. The present study shows that the fibre–soil interfacial characteristics were susceptible to not only overburden pressure, but also density and water content of soil. The higher the overburden pressure and density and the lower the water content, the tighter the fibre–soil interfacial bond and the larger the measurement range of optical fibre (Fig. 2a,b,f,g). Although the experimental data were limited, our findings indicate that an intimate connection between fibre and soil can hardly be ensured if the soil is loosely filled and/or highly saturated. In this context, optical fibres with anchorages are suggested to be adopted so that the interfacial bond between sensor and surrounding soil can be enhanced significantly^11^,^14^.

The failure of the fibre–soil interface has been found to be highly progressive during the deformation process of soil^11^,^27^. In the present study, the evolution of strain distribution captured by the BOTDA demodulator further confirmed this phenomenon. Compared with a previous study reported by Iten *et al*.^31^, our focus was on examining the validity of measured data during the course of progressive interface failure, which enabled us to identify and propose three working states of a soil-embedded strain sensing fibre (Fig. 4c). Earlier we performed small-scale optical fibre pull-out tests and employed classical structure–soil interaction models to describe the progressive failure process of the fibre–soil interface^23^,^24^,^25^. Unfortunately, because the optical fibres embedded in soil samples was so short (61.8 mm or 79.8 mm in length) that the BOTDA demodulator could hardly measure the strain distribution during testing, the proposed methodology was not strictly validated. In this study, the proposed model was verified by not only the pull-out force–displacement curve but also the distributions of strain along the optical fibre (Fig. 3a,c).

However, the proposed model has some limitations. First, the model was established under one-dimensional conditions (e.g. the fibre responded only to soil deformation in the longitudinal direction). In engineering practices, the deformation field of soil is fairly complicated. The fibre cannot keep straight if the transverse deformation of soil is considerable, which in turn makes it more challenging to assess the validity of measured soil strains^11^,^29^. Second, the effect of anchorages on the fibre–soil interaction was not considered. Although some practitioners have employed optical fibres with anchorages located at certain intervals (10 cm to 30 cm in laboratory models^11^,^14^, and 2 m in the field^11^,^32^) to improve the interfacial bond between fibre and soil, the underlying mechanism has seldom been investigated and remained largely unknown^11^,^30^. These two factors should be taken into consideration in further studies so that a more refined model can be established.

**Figure 1:**
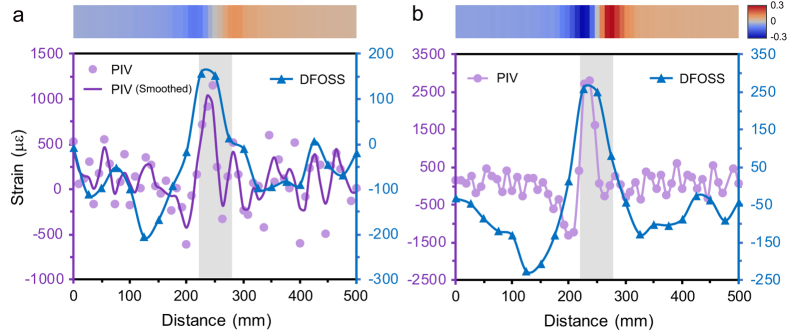
Results of an integrated distributed fibre optic strain sensing (DFOSS)- and photogrammetry-based study on the deformation behaviour of a small-scale sand foundation under surcharge loads. Comparison of soil strains along m–m’ obtained by DFOSS and particle image velocimetry (PIV) techniques under surcharge loads of 53.5 kPa (**a**) and 73.9 kPa (**b**), respectively. m–m’ is the installation locations of the optical fibres from a front view (Supplementary Fig. S1a). DFOSS strain data were averaged over two optical fibres placed in parallel. PIV data under the surcharge load of 53.5 kPa were smoothed. The boxes shaded in grey represent the footing. Heat maps display the horizontal displacements of soil along m–m’ (unit: pixel).

**Figure 2:**
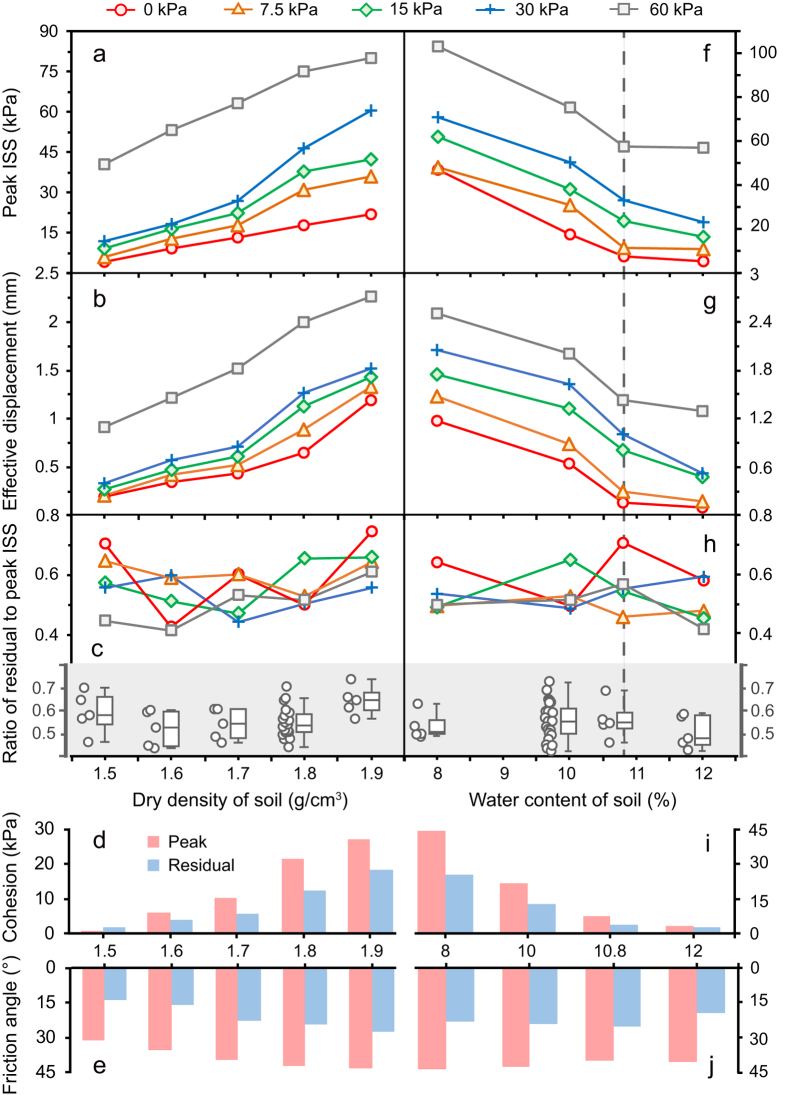
Influence of dry density and water content of soil on the fibre–soil interfacial behaviour. For each dry density or water content, overburden pressures (OPs) ranging from 0 kPa to 60 kPa were investigated. For each dry density, water content was 10%. For each water content, dry density was 1.8 g/cm^3^. Peak interfacial shear stress (ISS) **(a,f)**, effective displacement **(b,g)**, and ratio of residual to peak ISS **(c,h)** were inverted from the pull-out force–displacement curves (Supplementary Fig. S2) using an explicit model^25^. Boxplots in **(c,h)** depict all the data under each dry density or water content. Cohesion **(d,i)** and friction angle **(e,j)** were obtained from the ISS**–**OP relationships fitted with linear lines (Supplementary Fig. S4, and Supplementary Table 1). Grey dashed line indicates optimum water content of the soil.

**Figure 3:**
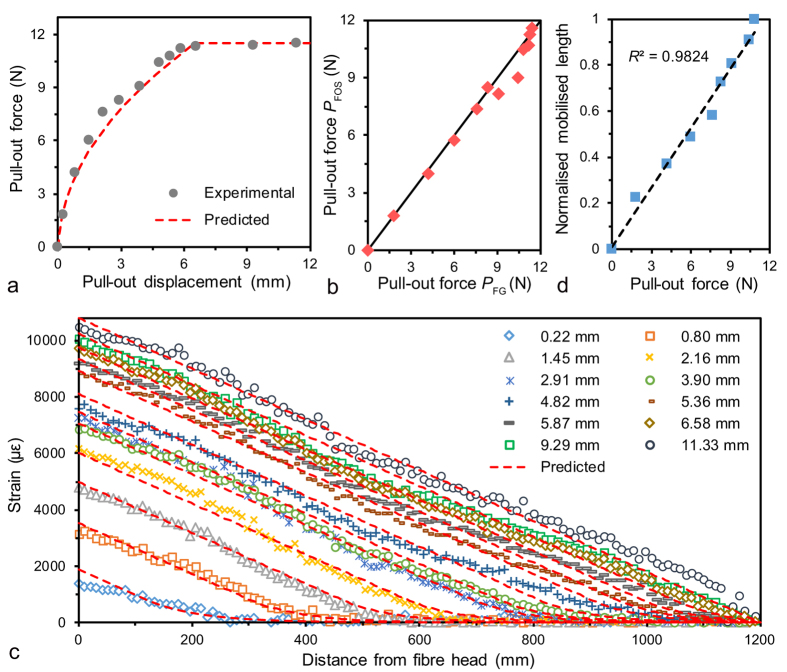
Evolution of strain distribution during progressive fibre–soil interface failure. **(a)** Curve of pull-out force versus pull-out displacement. **(b)** Correlation between pull-out forces measured by the force gauge (*P*_FG_) and those calculated using measured fibre optic strain data (*P*_FOS_). Solid line indicates 1:1 ratio. **(c)** Evolution of the strain distribution along the optical fibre under incremental displacements. Red dashed lines in **(a,c)** indicate results predicted by a fibre–soil interaction model proposed in this study. **(d)** Correlation between pull-out force and mobilised length (normalised). Dashed line indicates linear fits. Pull-out force in **(a,d)** refers to *P*_FG_.

**Figure 4:**
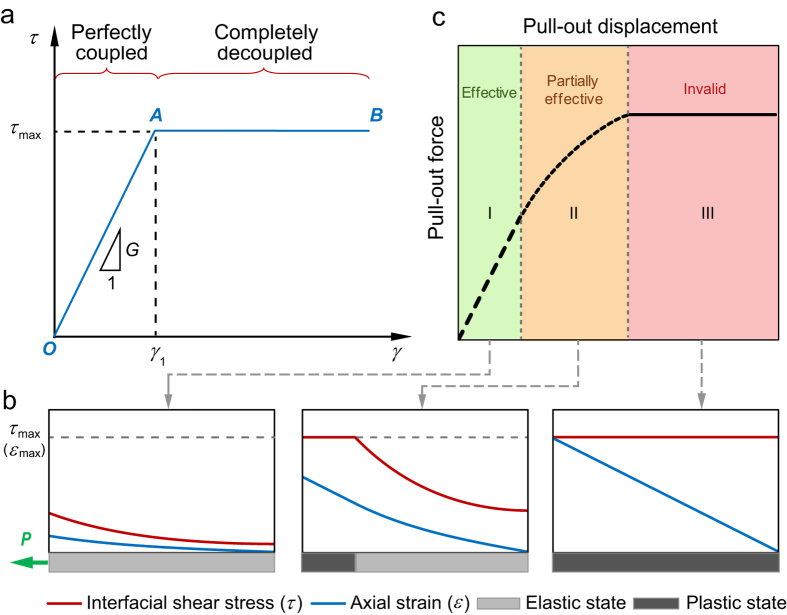
A simplified model proposed to describe the fibre–soil interfacial behaviour during progressive failure process. **(a)** Relationship between shear stress and shear strain for the fibre–soil interface. OA and AB denote elastic and plastic branches, respectively. **(b)** Evolution of the distribution of interfacial shear stress and axial strain along the strain sensing fibre during the failure process. **(c)** A typical pull-out force–displacement curve derived from the model. Three pull-out phases (i.e. Phases I, II and III) are marked, and three working states (i.e. effective, partially effective and invalid states) of a strain sensing fibre are shaded for clarity. “*P*” indicates pull-out force applied on the fibre head.

In the sand foundation model test, for the first time we used PIV technique to validate the fibre optic strain measurements. The strains measured by the BOTDA technique were found to be much smaller than those obtained using the PIV technique (Fig. 1), which were mainly attributed to the imperfect contact between the fibre and the soil. However, there were some other factors that could lead to this discrepancy. (1) The plane strain condition was not strictly controlled. This could be due to the disturbance of the embedded optical fibres to the sand foundation, the eccentricity of the applied loads, as well as the non-uniformity of the compacted foundation model. (2) The quality of the photographs was affected by the imperfect test condition and the image processing process also caused errors. The lighting condition was not as good as expected and the distortion of images could not be entirely eliminated, so the quality of the captured photographs was not perfect. In addition, calculation errors might accumulate when computing the strain field of the sand foundation from the displacement field. (3) The fibre optic strain measurements were highly dependent on the spatial resolution of the BOTDA demodulator. The soil strains were measured with a spatial resolution of 50 mm. While this value was only 1/10 of the length of the test tank, its influence on the measured strain could not be neglected. All these factors could bring about uncertainties to the measurements presented in this study, and should therefore be taken into consideration in further research.

The DFOSS technique is a rapidly evolving technique and has enormous potentialities in monitoring deformations and movements of earth structures; however, there are still many problems to be addressed. The issue of deformation compatibility between fibre and soil is such an obstacle that must be overcome. Collectively, the findings of our study may not only shed light on the fibre–soil interfacial behaviour, but also have important implications for interpreting fibre optic strain measurements and deploying a reliable DFOSS-based geotechnical monitoring project. Nevertheless, further studies are needed to elucidate the contribution of anchorages to the improvement of fibre–soil interfacial bond to enable wider application of DFOSS technique to soil deformation measurement and early warning of related geologic hazards.

## Materials and Methods

### Integrated DFOSS- and photogrammetry-based performance evaluation of a sandy soil foundation under surcharge loads

The tested fibre was a single-mode optical fibre fabricated by Suzhou Nanzee Sensing Co., Ltd., China. It was coated by a polyurethane jacket and the outer diameter was 1.2 mm. The soil was clean river sand, which was collected from the Yangtze River, Nanjing, China. The average grain size was 0.35 mm. The coefficients of uniformity and curvature were 1.61 and 1.06, respectively. The soil was classified as poorly graded sand (SP) according to the Unified Soil Classification System. The BOTDA demodulator was NBX-6050 produced by Neubrex Co., Ltd., Japan. The spatial resolution and the sampling interval were set as 50 mm and 25 mm, respectively. The camera used here was Canon EOS 600D. The footing, made of aluminium, was 50 mm in length, 100 mm in width and 2 mm in thickness. The loads were applied statistically using iron weights.

Schematic of the experiment setup is shown in Supplementary Fig. S1. The test tank was made of acrylic with internal dimensions of 500 mm long by 100 mm wide by 200 mm high. The thicknesses of the front and back walls were selected as 35 mm in order to avoid undesirable deformation under high pressures. The soil was compacted in five layers into the tank. The water content and dry density of the soil were kept at 10% and 1.60 g/cm^3^, respectively. The relative density of soil was 0.84, indicating the soil was dense. When the soil was compacted at the height of 50 mm, 83 mm, 116 mm, and 150 mm from the bottom of the tank, two optical fibres were laid symmetrically in the box, respectively. We note that because the soil deformations away from the footing were so small that the PIV analyses fluctuated markedly, here only the strains of soil at the height of 150 mm are presented and discussed, and only the fibres at this height are sketched in Fig. S1a. The fibres were pre-strained using small plastic discs glued onto side walls to allow compressive strains to be detected. Multi-loadings were applied on the footing up to a maximum of 73.9 kPa. Under each loading, photographs were taken by the camera placed at the front of the test tank, and strains along the optical fibres were measured by the BOTDA demodulator.

The photographs captured by the camera were analysed using the PIV technique^33^, through which soil strains (in με) and displacements (in pixel) were obtained. PIV data under the surcharge load of 53.5 kPa fluctuated, so we smoothed them using the “smooth” function in MATLAB (span = 0.1, method = loess).

### Examining the influence of water content and density of soil on fibre–soil interface

The optical fibre tested here was a single-mode fibre provided by Suzhou Nanzee Sensing Co., Ltd., China. It was coated by a hytrel jacket. The diameter of the optical fibre was 0.9 mm, and the Young’s modulus of the optical fibre was 1.75 GPa according to the results of standard uniaxial tensile tests. The soil used here was a poorly graded sand (SP) obtained from a construction site in Nanjing, China. The detailed physical and mechanical properties of the sand can be found in Zhang *et al*.^27^.

Pull-out tests were performed following the procedure proposed by Zhang *et al*.^27^. Of note, the diameter of the cutting ring was changed to 79.8 mm to ensure a longer bonding length and hence a better repeatability of the data. Dry densities of soil ranging from 1.5 g/cm^3^ to 1.9 g/cm^3^ and water contents ranging from 8% to 12% were investigated. For each dry density, the water content was kept at 10%; while for each water content, the dry density was kept at 1.8 g/cm^3^. OPs ranging from 0 kPa to 60 kPa were applied on all the test samples.

The obtained pull-out force–displacement curves (Supplementary Fig. S2) were interpreted using an explicit model^29^, through which various interfacial parameters were determined.

### Measuring the evolution of strain along the optical fibre

The tested optical fibre was 1200 mm in length and 2 mm in diameter, which was fabricated by Suzhou Nanzee Sensing Co., Ltd., China. The material of the jacket was polyurethane. The Young’s modulus was 0.34 GPa, which was determined based on the results of standard uniaxial tensile tests. The soil and the BOTDA demodulator used here were the same as those used in the foundation test.

Schematic diagram of the experimental apparatus is shown in Supplementary Fig. S5. The test tank was made of aluminium with internal dimensions of 1500 mm long by 200 mm wide by 200 mm high. A 60 mm-diameter hole was drilled on the front wall. The soil was compacted in five layers into the tank. The water content and dry density of the soil were kept at 5% and 1.53 g/cm^3^, respectively. A 200-mm free segment of fibre was left in the vicinity of the front wall to eliminate the boundary effect. Pull-out displacements were applied by an electric motor. The pull-out forces and displacements were measured by a force gauge and a dial gauge, respectively. Another dial gauge was pointed at the back wall to ensure that the test tank was stationary. A maximum displacement of 11.33 mm was applied and 12 sets of strain data were obtained.

## Additional Information

**How to cite this article**: Zhang, C.-C. *et al*. Role of the interface between distributed fibre optic strain sensor and soil in ground deformation measurement. *Sci. Rep*. **6**, 36469; doi: 10.1038/srep36469 (2016).

**Publisher’s note**: Springer Nature remains neutral with regard to jurisdictional claims in published maps and institutional affiliations.

## Supplementary Material

Supplementary Information

## References

[b1] Lienhart W. Case studies of high-sensitivity monitoring of natural and engineered slopes. J. Rock Mech. Geotech. Eng. 7, 379–384 (2015).

[b2] Horiguchi T. & Tateda M. Optical-fiber-attenuation investigation using stimulated Brillouin scattering between a pulse and a continuous wave. Opt. Lett. 14, 408–410 (1989). 19749936 10.1364/ol.14.000408

[b3] Rogers A. J. & Handerek V. A. Frequency-derived distributed optical-fiber sensing: Rayleigh backscatter analysis. Appl. Opt. 31, 4091–4095 (1992). 20725386 10.1364/AO.31.004091

[b4] Bao X., Dhliwayo J., Heron N., Webb D. J. & Jackson D. A. Experimental and theoretical studies on a distributed temperature sensor based on Brillouin scattering. J. Lightw. Technol. 13, 1340–1348 (1995).

[b5] Thévenaz L. Brillouin distributed time-domain sensing in optical fibers: State of the art and perspectives. Front. Optoelectron. China 3, 13–21 (2010).

[b6] Dong Y., Chen L. & Bao X. Time-division multiplexing-based BOTDA over 100 km sensing length. Opt. Lett. 36, 277–279 (2011). 21263525 10.1364/OL.36.000277

[b7] Soto M. A., Taki M., Bolognini G. & Di Pasquale F. Simplex-coded BOTDA sensor over 120-km SMF with 1-m spatial resolution assisted by optimized bidirectional Raman amplification. IEEE Photonics Technol. Lett. 24, 1823–1826 (2012).

[b8] Loranger S., Gagné M., Lambin-Iezzi V. & Kashyap R. Rayleigh scatter based order of magnitude increase in distributed temperature and strain sensing by simple UV exposure of optical fibre. Sci. Rep. 5, 11177 (2015). 26077365 10.1038/srep11177 PMC4650692

[b9] Ho Y. T., Huang A. B. & Lee J. T. Development of a fibre Bragg grating sensored ground movement monitoring system. Meas. Sci. Technol. 17, 1733 (2006).

[b10] Habel W. R. & Krebber K. Fiber-optic sensor applications in civil and geotechnical engineering. Photonic Sens. 1, 268–280 (2011).

[b11] Iten M. Novel applications of distributed fiber-optic sensing in geotechnical engineering. PhD thesis, Federal Institute of Technology in Zurich (2011).

[b12] Sun Y. J. . Distributed acquisition, characterization and process analysis of multi-field information in slopes. Eng. Geol. 182, 49–62 (2014).

[b13] Zeni L. . Brillouin optical time-domain analysis for geotechnical monitoring. J. Rock Mech. Geotech. Eng. 7, 458–462 (2015).

[b14] Zhu H. H., Shi B., Zhang J., Yan J. F. & Zhang C. C. Distributed fiber optic monitoring and stability analysis of a model slope under surcharge loading. J. Mt. Sci. 11, 979–989 (2014).

[b15] Klar A. . Distributed strain measurement for pile foundations. Proc. Inst. Civil Eng.-Geotech. Eng. 159, 135–144 (2006).

[b16] Lu Y., Shi B., Wei G. Q., Chen S. E. & Zhang D. Application of a distributed optical fiber sensing technique in monitoring the stress of precast piles. Smart Mater. Struct. 21, 115011 (2012).

[b17] Lanticq V. . Soil-embedded optical fiber sensing cable interrogated by Brillouin optical time-domain reflectometry (B-OTDR) and optical frequency-domain reflectometry (OFDR) for embedded cavity detection and sinkhole warning system Meas. Sci. Technol. 20, 034018 (2009).

[b18] Linker R. & Klar A. Detection of sinkhole formation by strain profile measurements using BOTDR: Simulation study. J. Eng. Mech. doi: 10.1061/(ASCE)EM.1943-7889.0000963 (2015).

[b19] Buchoud E. . Quantification of submillimeter displacements by distributed optical fiber sensors. IEEE Trans. Instrum. Meas. 65, 413–422 (2016).

[b20] Iten M., Puzrin A. M. & Schmid A. Landslide monitoring using a road-embedded optical fiber sensor. Proc. SPIE 6933, 693315 (2008).

[b21] Ansari F. & Yuan L. Mechanics of bond and interface shear transfer in optical fiber sensors. J. Eng. Mech. 124, 385–394 (1998).

[b22] Yuan L. B. & Zhou L. M. Sensitivity coefficient evaluation of an embedded fiber-optic strain sensor. Sens. Actuator A-Phys. 69, 5–11 (1998).

[b23] Leung C., Wang X. & Olson N. Debonding and calibration shift of optical fiber sensors in concrete. J. Eng. Mech. 126, 300–307 (2000).

[b24] Li H. N., Zhou G. D., Ren L. & Li D. S. Strain transfer coefficient analyses for embedded fiber Bragg grating sensors in different host materials. J. Eng. Mech. 135, 1343–1353 (2009).

[b25] Wang B. J., Li K., Shi B. & Wei G. Q. Test on application of distributed fiber optic sensing technique into soil slope monitoring. Landslides 6, 61–68 (2009).

[b26] Picarelli L. . Performance of slope behavior indicators in unsaturated pyroclastic soils. J. Mt. Sci. 12, 1434–1447 (2015).

[b27] Zhang C. C., Zhu H. H., Shi B. & She J. K. Interfacial characterization of soil-embedded optical fiber for ground deformation measurement. Smart Mater. Struct. 23, 095022 (2014).

[b28] Zhang C. C., Zhu H. H., She J. K., Zhang D. & Shi B. Quantitative evaluation of optical fiber/soil interfacial behavior and its implications for sensing fiber selection. IEEE Sens. J. 15, 3059–3067 (2015).

[b29] Zhu H. H., She J. K., Zhang C. C. & Shi B. Experimental study on pullout performance of sensing optical fibers in compacted sand. Measurement 73, 284–294 (2015).

[b30] ASTM F3079-14. Standard Practice for Use of Distributed Optical Fiber Sensing Systems for Monitoring the Impact of Ground Movements During Tunnel and Utility Construction on Existing Underground Utilities (ASTM International, West Conshohocken, PA, 2014).

[b31] Iten M. . Study of a progressive failure in soil using BEDS. Proc. SPIE 7503, 75037S-4 (2009).

[b32] Suo W. . Development and application of a fxed-point fber-optic sensing cable for ground fssure monitoring. J. Civ. Struct. Health Monit. 6, 715–724 (2016).

[b33] Stanier S. A., Blaber J., Take W. A. & White D. Improved image-based deformation measurement for geotechnical applications. Can. Geotech. J. 53, 727–739 (2016).

