# Supplementary material for: Role of the interface between distributed fibre optic strain sensor and soil in ground deformation measurement
Source: Sci Rep. 2016 Nov 9;6:36469. doi: 10.1038/srep36469 (PMC5101507; doi:10.1038/srep36469)
Supplement: Supplementary Information [file srep36469-s1.pdf]

<Supplementary Information>

Role of the interface between distributed fibre optic strain sensor and soil in soil deformation measurement

Cheng-Cheng Zhang[1], Hong-Hu Zhu[1,2,*], and Bin Shi[1]

[1]School of Earth Sciences and Engineering, Nanjing University, Nanjing 210023, China

[2]Department of Engineering, University of Cambridge, Cambridge CB2 1PZ, United Kingdom

*zhh@nju.edu.cn

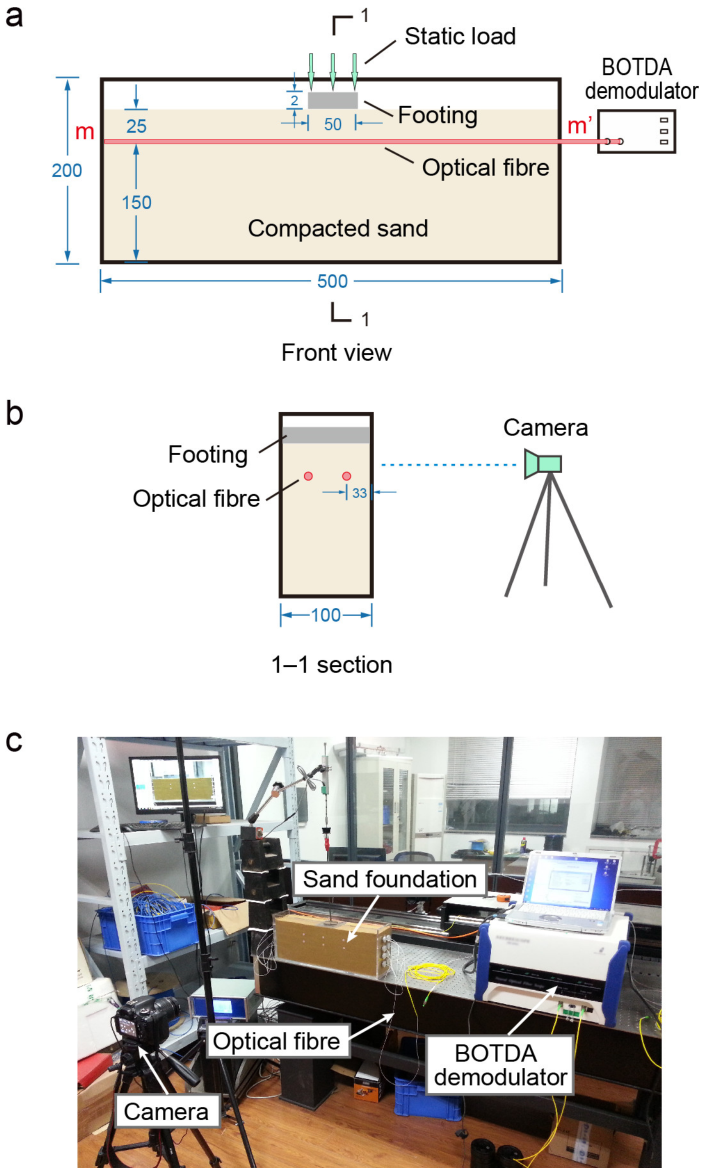

Supplementary Figure S1. Experimental setup of an integrated distributed fibre optic strain sensing (DFOSS)- and photogrammetry-based study on the deformation behaviour of a small-scale sand foundation under surcharge loads. (a–b) Schematic diagram of the experiment setup (unit: mm). **(c)** A photograph showing the prepared sand foundation to be tested.

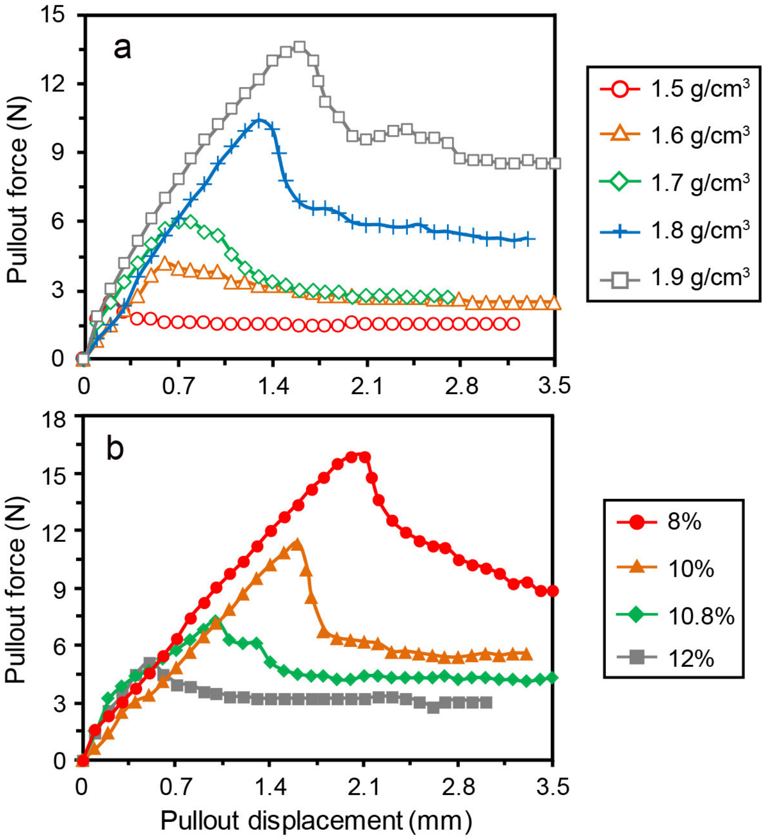

Supplementary Figure S2. Selected pull-out force–displacement curves under various dry densities (a) and water contents of soil (b). Overburden pressure (OP) was 30 kPa. For each dry density, water content was 10%. For each water content, dry density was 1.8 g/cm^3.

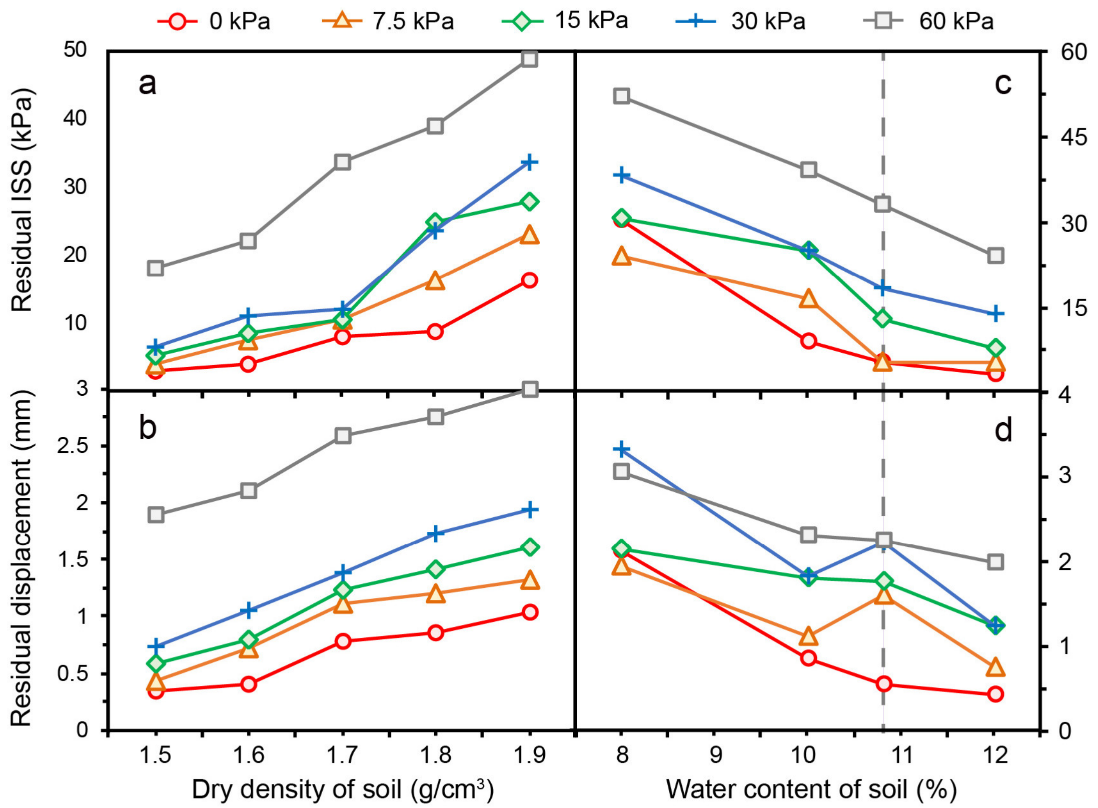

Supplementary Figure S3. Influence of dry density and water content of soil on the residual interfacial shear stress (ISS) and residual displacement. For each dry density or water content, OPs ranging from 0 kPa to 60 kPa were investigated. Residual ISS **(a,c)** and residual displacement **(b,d)** were inverted from the pull-out force–displacement curves (Supplementary Fig. S2). Grey dashed line indicates optimum water content of the soil.

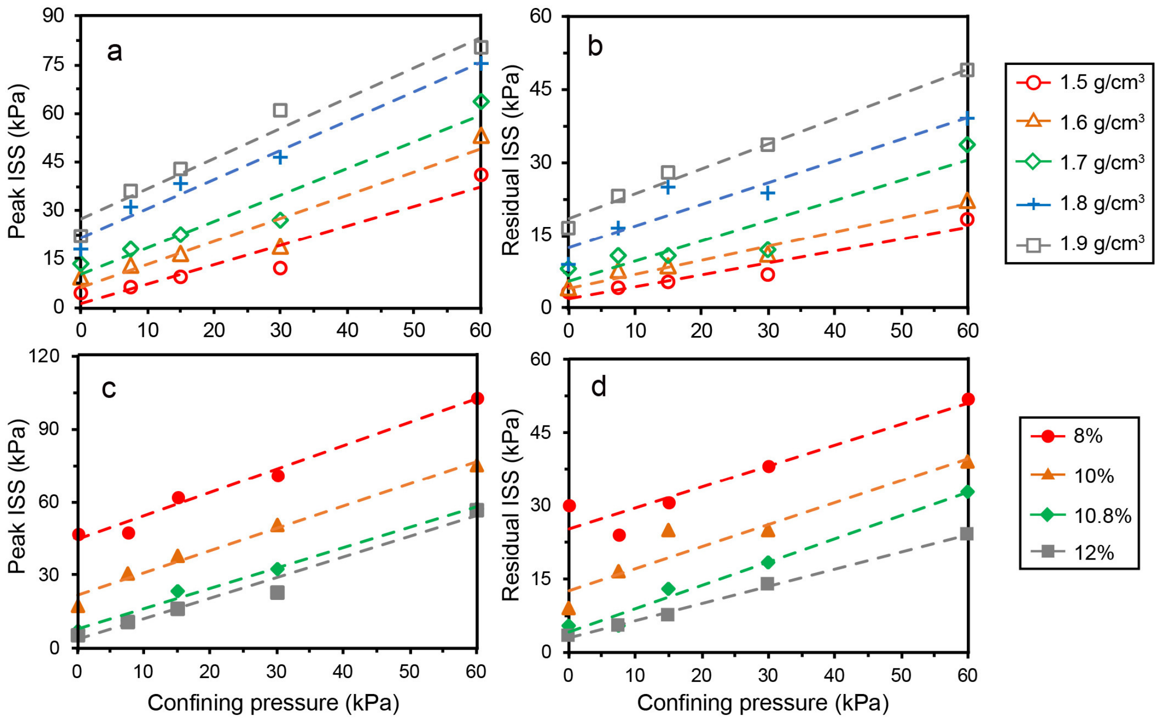

Supplementary Figure S4. ISS–OP relationships under various dry densities and water contents of soil. Linear fits made to each dry density **(a,b)** or water content **(c,d)** of soil are displayed as dashed lines. Fitted lines, summarized in Supplementary Table S1, were used to obtain the cohesions and friction angles of the fibre–soil interface.

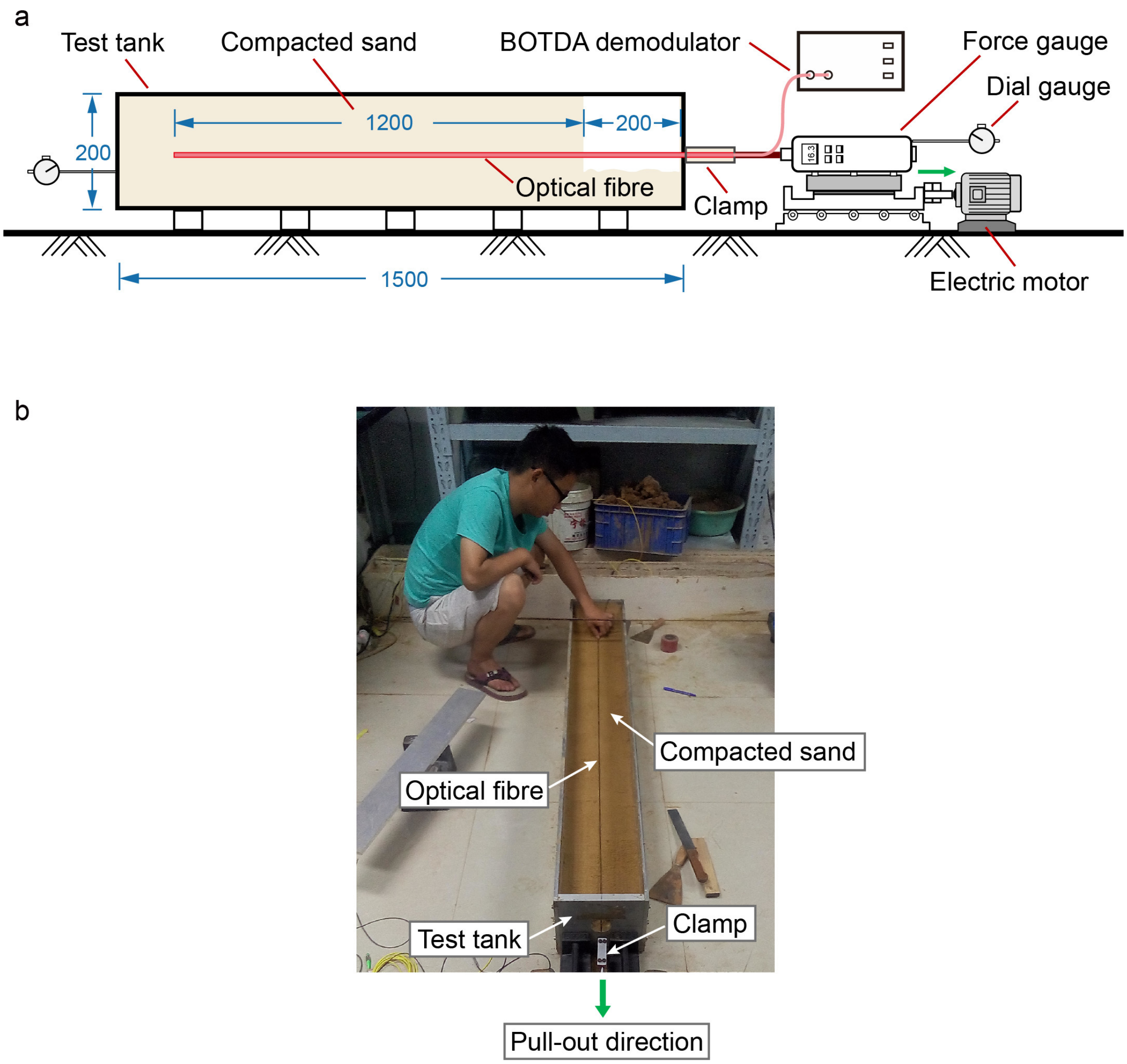

Supplementary Figure S5. Setup of the experiment to measure strain distribution evolution during progressive fibre–soil interface failure. (a) Schematic diagram of the experimental apparatus (unit: mm). **(b)** Layout of the optical fibre in the test tank.

Dry density (g/cm^3)	Water content (%)	Peak ISS (kPa)			Residual ISS (kPa)		
		a	*b*	R^2	*a*	*b*	R^2
1.5	10	0.603	0.975	0.915	0.250	1.83	0.929
1.6	10	0.715	5.98	0.913	0.288	4.16	0.974
1.7	10	0.820	10.4	0.945	0.415	5.64	0.870
1.8	10	0.900	21.5	0.981	0.448	12.4	0.898
1.9	10	0.937	27.2	0.966	0.514	18.4	0.986
1.8	8	0.962	44.6	0.982	0.428	25.3	0.899
1.8	10	0.913	22.0	0.983	0.452	12.6	0.909
1.8	10.8	0.839	7.71	0.990	0.476	4.19	0.981
1.8	12	0.841	3.60	0.973	0.353	2.78	0.998

Supplementary Table S1. ISS–OP relationships fitted with linear lines. $y = a + bx$. R^2 denotes coefficient of determination.

Supplementary Methods

The optical fibre–soil interface follows the elasto–plastic shear stress–strain constitutive relations:

$$\tau(x) = \begin{cases} G\gamma(x) & (0 \le \gamma < \gamma_1) \\ \tau_{\max} & (\gamma \ge \gamma_1) \end{cases} \tag{S1}$$

where τ and γ are the interfacial shear stress and strain, respectively; γ_1 is the interfacial shear strain corresponding to the peak ISS, $\tau_{\max}$; and G is the shear stiffness of the optical fibre–soil interface. By combining equation (S1) with classical equations of the equilibrium of an infinitesimal fibre element, the axial strain, interfacial shear stress and displacement for each of the three phases can be derived[1].

(1) Pure elastic stage (Phase I)

In this stage, the axial strain, interfacial shear stress and displacement are:

$$\varepsilon(x) = \frac{P}{AE}\frac{\sinh\beta(L-x)}{\sinh\beta L} \tag{S2}$$

$$\tau(x) = -\frac{\beta P}{\pi D}\frac{\cosh\beta(L-x)}{\sinh\beta L} \tag{S3}$$

$$u(x) = \frac{\beta P}{\pi D G^*}\frac{\cosh\beta(L-x)}{\sinh\beta L} \tag{S4}$$

where P is the pull-out force applied on the fibre head; D, L, A and E are the diameter, length, cross-sectional area and Young's modulus of the optical fibre, respectively; G^* is a shear coefficient of the fibre–soil interface defined as $G^* = 2G/h$; h is the thickness

of the shearing band along the fibre; and β is a coefficient defined by $\beta = \sqrt{4G^{*} / ED}$.

Let $x = 0$ in equation (S4), we get:

$$P = \frac{\pi D G^{*}}{\beta} \tanh(\beta L) u_{0} \tag{S5}$$

where $u_{0} = u(0)$ is the displacement at the fibre head.

(2) Elasto–plastic (Phase II)

In Phase II, the distributions of axial strain, interfacial shear stress and displacement in the elastic zone ($L_{p} \leq x \leq L$) are similar to those in Phase I:

$$\varepsilon(x) = \frac{F_{T}}{EA} \frac{\sinh \beta(L - x)}{\sinh \beta(L - L_{p})} \tag{S6}$$

$$\tau(x) = -\frac{\beta F_{T}}{\pi D} \frac{\cosh \beta(L - x)}{\sinh \beta(L - L_{p})} \tag{S7}$$

$$u(x) = -\frac{\beta F_{T}}{\pi D G^{*}} \frac{\cosh \beta(L - x)}{\sinh \beta(L - L_{p})} \tag{S8}$$

where L_{p} is the length of the plastic zone; and F_{T} is the tensile force at the transition point between the elastic and plastic zones:

$$F_{T} = \frac{\pi D \tau_{\max} \tanh \beta(L - L_{p})}{\beta} \tag{S9}$$

In the plastic zone ($0 \leq x \leq L_{p}$), $\tau(x) = \tau_{\max}$. The axial strain and displacement are:

$$\varepsilon(x) = \frac{4\tau_{\max}}{DE}(L_{p} - x) + \frac{4D\tau_{\max} \tanh \beta(L - L_{p})}{DE\beta} \tag{S10}$$

$$u(x) = \frac{2\tau_{\max}}{ED}(L_{p}{}^{2} - x^{2}) - \frac{P}{EA}(L_{p} - x) - \frac{\tau_{\max}}{G^{*}} \tag{S11}$$

Let $x = 0$ in equation (S11), we get:

$$P = -\frac{AE}{L_p}(u_0 + \frac{\tau_{\max}}{G^*}) + \frac{\pi D}{2} L_p \tau_{\max} \tag{S12}$$

(3) Pure plastic phase (Phase III)

In Phase III, interfacial shear stress equals $\tau_{\max}$ as well. The axial strain is:

$$\varepsilon(x) = \frac{4}{DE}\tau_{\max}(L - x) \tag{S13}$$

Assuming that the displacement at the fibre head is u_0', the distribution of displacement is:

$$u(x) = u_0' + \frac{2\tau_{\max}}{ED}(2Lx - x^2) \tag{S14}$$

Let $x = 0$ in equation (S13), we get:

$$P = \pi D \tau_{\max} L \tag{S15}$$

Supplementary Reference

1. Zhang, C. C., Zhu, H. H., Shi, B., Wu, F.D. & Yin, J.H. Experimental investigation of pullout behavior of fiber-reinforced polymer reinforcements in sand. *J. Compos. Constr.* **19,** 04014062 (2015).
